# Nutrition supplementation plus standard of care versus standard of care alone or standard of care plus unconditional cash transfer in the prevention of chronic malnutrition in Southern Angola: study protocol for the MuCCUA study, a cluster randomized controlled trial

**DOI:** 10.1186/s12889-024-17858-7

**Published:** 2024-02-10

**Authors:** E Custodio, Z Herrador, E Trigo, M Romay-Barja, F Ramirez, I Aguado, E Iraizoz, A Silva-Gerardo, ML Faria, R Martin-Cañavate, T Marques, A Vargas, A Gómez, I Molina

**Affiliations:** 1 Centro de Investigación Biomédica en Red de Enfermedades Infecciosas, (CIBERINFEC), Instituto de Salud Carlos III, Madrid, 28029 Spain; 2 Centro Nacional de Medicina Tropical, Instituto de Salud Carlos III, Avda. Monforte de Lemos, 3, Madrid, 28029 Spain; 3 Centro Nacional de Epidemiología, Instituto de Salud Carlos III, Avda. Monforte de Lemos, 3, Madrid, 28029 Spain; 4 Tropical Medicine Unit Vall d’Hebron-Drassanes, Infectious Diseases Department, Vall d’Hebron University Hospital, PROSICS Barcelona, Barcelona, 08035 Spain; 5 Action Against Hunger Spain, C/Duque de Sevilla 3, Madrid, 28002 Spain; 6 Faculdade de Medicina da Universidade Mandume Ya Ndemufayo, Bairro Comercial, Avenida Hoji Ya Haenda nº30, caixa postal 201, Lubango, Huíla Angola; 7 Fundo Apoio Social-Local Development Institute, Avenida Pedro de Castro Vandunem, Luanda, Angola

**Keywords:** Stunting, Children, Pregnant women, Cash transfer, Food supplementation, SQ-LNS, Angola, Randomised control trial, Research protocol

## Abstract

**Background:**

Chronic malnutrition is a condition associated with negative impacts on physical and cognitive development. It is multi-causal and can start very early in life, already in utero, thus it is especially challenging to find appropriate interventions to tackle it. The government of Angola is implementing a standard of care program with potential to prevent it, and the provision of cash transfers and the supplementation with small quantity lipid-based nutrients (SQ-LNS) are also promising interventions. We aimed to evaluate the impact of the standard of care program alone and of the standard of care plus a cash transfer intervention in the lineal growth of children less than 2 years old and compare it to the effectiveness of a nutrition supplementation plus standard of care program in Southern Angola.

**Methods/design:**

The three-arm parallel cluster randomised controlled trial is set in four communes of Huila and Cunene provinces. Clusters are villages or neighbourhoods with a population around 1075 people. A total of twelve clusters were selected per arm and forty pregnant women are expected to be recruited in each cluster. Pregnant women receive the standard of care alone, or the standard of care plus unconditional cash transfer or plus nutritional supplementation during the first 1000 days, from pregnancy to the child reaching 24 months. The primary outcome is the prevalence of stunting measured as height-for-age Z-score (HAZ) < -2 in children below 2 years. Impact will be assessed at 3, 6, 12, 18 and 24 months of children’s age. Secondary outcomes include mortality, morbidity, caring, hygiene and nutrition behaviours and practices, and women and children’s dietary diversity. Quantitative data are also collected on women’s empowerment, household food security, expenditure and relevant clinical and social events at baseline, endline and intermediate time points.

**Discussion:**

The results will provide valuable information on the impact of the standard of care intervention alone as well as combined with an unconditional cash transfer intervention compared to a nutrition supplementation plus standard of care intervention, carried out during the first 1000 days, in the children´s growth up to 2 years and related outcomes in Southern Angola.

**Trial registration:**

Clinical Trials NCT05571280. Registered 7 October 2022.

## Introduction

### Background and rationale

Stunting or low height-for-age among children under five years of age is an indicator of growth faltering associated with chronic malnutrition, a condition that can have important negative impacts on subsequent physical and cognitive development [1].

According to the most recent estimates, there are still 149 million children under five years of age affected by stunting globally (accounting for 22% of all children under five years) and Africa is the region showing the highest prevalence (30.7%) [2].

The determinants of stunting and other forms of malnutrition, as well as the interventions needed to combat them were summarized in the 2008 and 2013 Lancet Series on maternal and child nutrition [3, 4]. The most direct determinants or *immediate causes* are inappropriate dietary intake and diseases that are influenced by the *intermediate causes*, grouped in caring practices, food security, and access to water, sanitation and health services. These in turn are grounded on the *basic or structural causes* comprising poverty, education, and lack of basic community resources, among others [5]. The Series also outlined several effective interventions mostly tackling immediate causes, the so-called *nutrition-specific* interventions [6], but also emphasized the need to go beyond and address the intermediate and basic causes through the *nutrition-sensitive* interventions [7, 8]. These latter interventions have a nutrition goal but are implemented from different sectors such education, water and sanitation, agriculture or social safety nets, among others.

In relation to stunting more specifically, the evidence compiled highlighted the need to focus on prevention, with interventions targeting pregnancy and the first two years of children’s lives, known as the “First 1000 days” period [9]. This is related to the fact that stunting begins very early in life (in utero) and generally continues during the first two postnatal years, with most of the decline in length-for-age occurring during the complementary feeding period, between 6 and 24 months of age [10]. Some of the nutrition specific interventions recommended for the first 1000 days period were: preventive strategies for malaria in pregnancy, the promotion of early and exclusive breastfeeding, vitamin A supplementation in children 6 to 59 months of age, and management of acute malnutrition (moderate and severe), and among the nutrition sensitive interventions, “WASH interventions” showed positive results [7].

In 2021 the evidence of the Lancet Series was expanded by the revision of effectiveness of newer interventions [11]. Among the *nutrition-specific* interventions stood out the supplementation with small-quantity lipid-based nutrients (SQ-LNS) for the improvement of child growth [12], and for stunting reduction among newborns by the provision of SQ-LNS to the pregnant women [13]. The positive effects of SQLNS on growth has been further validated by a meta-analysis that provided evidence of this positive effect in a variety of contexts, recommending to policy makers its inclusion in intervention packages aimed at reducing stunting or wasting [14].

In relation to *nutrition-sensitive* interventions there is emerging interest in the effectiveness of cash-based transfers for preventing malnutrition, as it is a public health strategy that has proven effectiveness on improving household food security [8], child health outcomes [15], and children’s growth [16].

In Angola, the prevalence ofstunting is 38% with large disparities across geographical areas of the country and within diverse agro-ecological zones [17]. Moreover, persistent food security and nutritional crisis caused by cyclical phenomena of droughts and floods have affected the southern provinces of Angola in recent years, resulting in around 50% of communes classifiedas *Crisis* levels of food insecurity and *Very high* levels of malnutrition [18].

The goal of this study is to generate evidence on the effectiveness of *nutrition-specific* and *nutrition-sensitive* interventions in the prevention of growth retardation, in Southern Angola.

More specifically, the MuCCUA study (“Mother and Child Chronic Undernutrition in Angola” Study) aims to assess the effectiveness of a standard of care program alone or plus a cash transfer intervention on the prevention of growth retardation during the first 1000 days of life, and compare each of them with the effectiveness of a nutrition supplementation plus standard of care intervention, in two Southern provinces of Angola (Huila and Cunene).

The study is part of the project Crescer, one of the components of the FRESAN (Fortalecimento da Resiliência e da Segurança Alimentar e Nutricional em Angola) program, an EU initiative to strength resilience and food and nutrition security in Southern Angola.

## Methods/design

The Crescer project is implemented by a consortium consisting of five partners; two Angolan institutions (Universidade Mandume Ya Ndemufayo and Fundo de Apoio Social) and three from Spain: the Hospital Universitari Vall d’Hebron Research Institute (consortium’s coordinator), the Instituto de Salud Carlos III and Action Against Hunger Spain. The European Union funds it.

### Study design

The study is conceived as a community trial, controlled, open, non-inferiority, randomized by clusters, with three intervention arms grouped by blocks (communes) in parallel groups, in two provinces of Southern Angola, Huíla and Cunene. The cluster allocation ratio is 1:1:1.

The study is prospective, pregnant women will be recruited and followed along their newborns until the children turn 2 years old.

### Study setting

The MuCCUA trial is set in two provinces of Southern Angola: Huila and Cunene. The most recent official data on malnutrition estimates that the prevalence ofstunting in selected areas of Huila and Cunene provinces is 50% and 37% respectively, and the prevalence of acute malnutrition around 11% in both provinces for children under five years of age. In addition, the prevalence of stunting in children below 2 years of age raises to 51.8% in Huila and to 44.6% in Cunene [19].

**Fig. 1:**
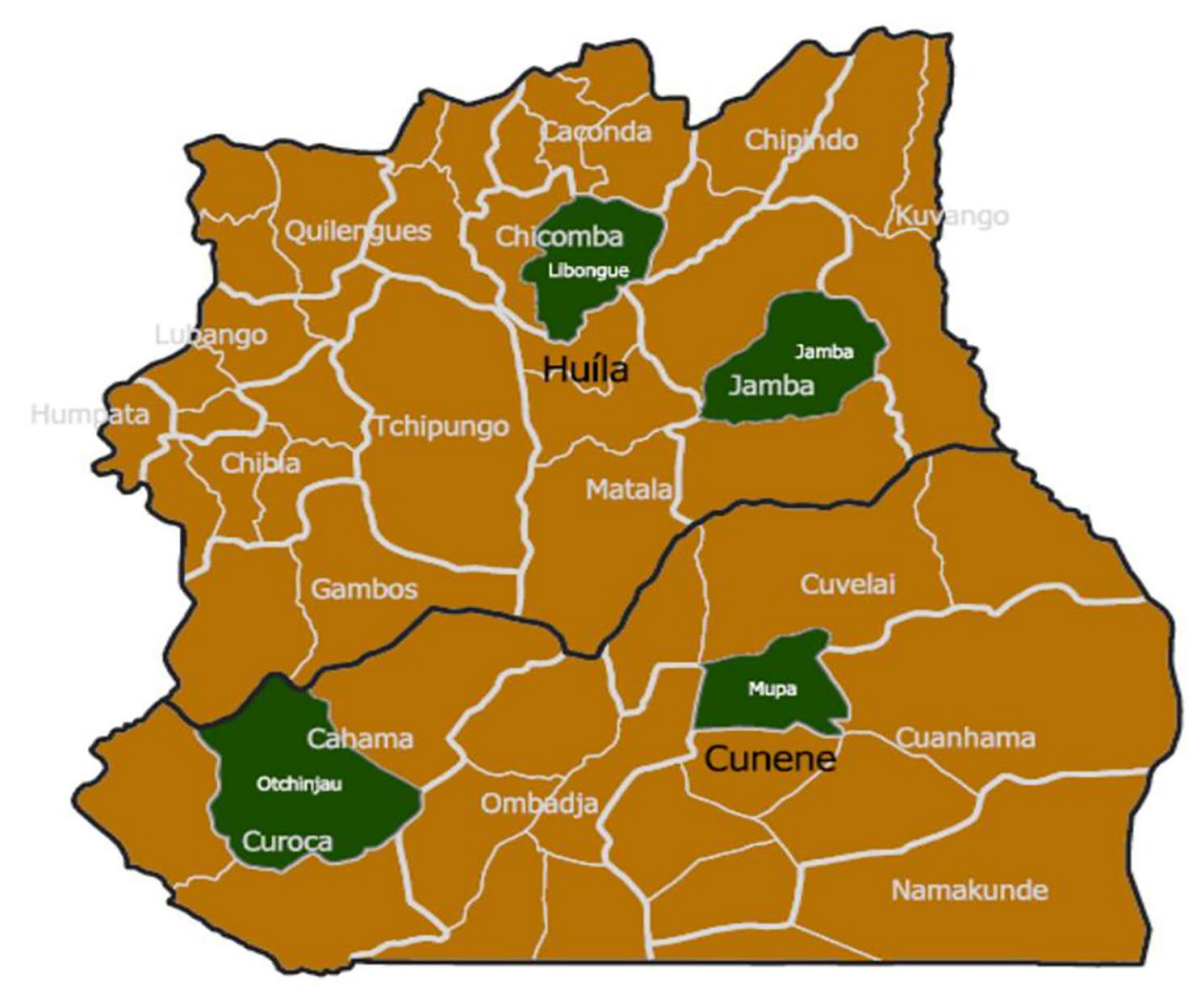
Study communes (green) within Huila and Cunene provinces

The trial is carried out in these two provinces (*admin 1* level). Two municipalities (*admin 2* level) per province were selected with one commune (*admin 3 l*evel) per municipality (total two communes per province); the commune of Libongue (Chicomba municipality) and the commune of Jamba (municipality of Jamba) in the province of Huíla. The commune of Otchinjau (municipality of Cahama) and the commune of Mupa (municipality of Cuvelai) in Cunene province. See Fig. 1.

### The interventions

The standard of care intervention is provided at cluster level, the cash transfer, and nutrition supplements interventions at the individual level.

#### Standard of care arm (SoC arm)

The new national policy for community development of the Ministry of Territorial Administration of the Republic of Angola foresees the implementation of a standard of care program to be delivered by a new figure, the ADECOS (Agentes de Desenvolvimento Comunitário e Sanitário) through the Institute of Local Development-Social Support Fund (FAS), included in the Health Community Workers classification. The ADECOS emerged as one of the strategies with more potential to address cost-effectively the scarcity of health human resources in remote areas, as they should be able to provide culturally competent services to improve accessibility to primary health care and community awareness in water, hygiene and sanitation (WASH), nutrition, and health related good practices.

This policy is in different implementation phases across the country by the Government of Angola, but the Southern provinces have not yet started its implementation at the health care level. Thus, in the study areas of the provinces of Huila and Cunene, the Crescer project financed its implementation through the FAS, one of the partners of the Crescer consortium.

The activities to be developed by the ADECOS can be summarized in two types:

Health promotion activities: including bi weekly home visits and quarterly community activities to promote adequate management of acute malnutrition and promotion of appropriate caring and feeding practices with special emphasis on breastfeeding, and sensitization sessions to promote adequate hygiene and sanitation.

Preventive pharmacological activities: these are conducted quarterly and include malaria prophylaxis in pregnant women, deworming in pregnant women and children between 12 and 59 months, and vitamin A supplementation in children 6 to 24 months of age.

The standard of care includes in its package many of the evidence-based interventions to tackle malnutrition identified in the 2013 Lancet Series [7] and may have an important impact on stunting in a population as deprived as the one in our study, although up to date, and as far as we know, there is no evidence on this effect.

#### Standard of care + nutritional supplementation arm (SoC + NS arm)

Communities allocated to this arm receive the *Standard of care* intervention plus a nutritional supplementation to families with at least one pregnant woman. Nutritional supplementation consists of a daily intake of small quantity lipid based nutrients supplement (SQ-LNS) for the pregnant women and their newborn children and a complementary food ration for their families, as described below.

- Individual ration of SQ-LNS: Nutriset Enov’ Mum™ for pregnant and lactating women until their new-born turns 6 months (1 sachet of 20 grs/day) and Nutriset Enovnutributter ®+ for the newborn children after their 6th month and until the child turns 24 months of age (1 sachet of 20 grs/ day).
- Complementary family food ration: basket of locally produced staple foods that complement the usual diet (300 Kcal/person/day). The caloric distribution of the basket will be 45% of cereals (corn meal - carbohydrate), 30% of legumes (beans - vegetable protein) and 25% of oil (soybean oil - fat). In addition, 1 kg of iodized salt will be provided.

The individual ratios of SQ-LNS will be distributed to households by the local ADECOS every two weeks, and the family ration by the project distribution team every three months in the central distribution point.

Out of the three interventions assessed, this intervention has the greatest evidence of its effect on stunting. Dewey meta-analysis showed that SQ-LNS provision led to a 12% stunting reduction (relative average) [14]. This arm will be usedas a comparator as it serves the primary purpose of the trial, and it fulfils the criteria of acceptability, feasibility, formidability and relevance required to be a good comparator [20].

#### Standard of care + cash transfer arm (SoC + CT arm)

Communities allocated to this arm will receive the *Standard of care* intervention plus an unconditional cash transfer given to families with at least one pregnant woman as describedbelow. The pregnant woman, as the main participant, is defined as the cash transfer recipient.

A total of 13,855 kwanzas per month is delivered to families with four or more members living in the household during the study period, and 10,855 kwanzas per month to families with less than four members living in the household during the period of study. The minimum inter professional salary in Angola at the time being is set at 35,000 kwanzas per month.

The amount is delivered in cash with unconditional format. The cash transfer will be distributed to the recruited women or persons authorized by her every three months in a central point of distribution.

### Study participants

The study population are pregnant women over 16 years of age living in the selected clusters, and their newborn children. The target population of the interventions are all the members living in the household where the pregnant woman lives. In the study area, a household or*agregado familiar* is defined as the group of people, with or without kinship relationships, that usually live together under the same roof, and for at least 6 months (or less than 6 months, but with the intention of staying in the residence for the next 6 months), and share food and/or other vital needs, and their house.

All pregnant women living in the selected cluster can be included in the study, up to a maximum of 40 per cluster.

### Sample size

We used the results from the most recent SMART survey [19] to determine the chronic malnutrition prevalences for the sampling computations, assuming an overall stunting prevalente in children under 2 years of 47,5%. We estimate an expected effect of the nutritional supplementation plus standard of care intervention to reduce stunting by 9.8%, and the same (9.8%) for the experimental arms (standard of care alone or standard of care plus cash transfer arms). Although the literature provides lower effects of stunting reduction for SQLNS interventions [14], we have assumed a higher effect due to several factors including that the SQLNS individual supplementation starts during the mothers pregnancy and is continued until the age of 2 years of the child, and is complemented with a food family ratio at household level. Moreover, the three arms involve the implementation of an optimized standard of care at community level that targets many of the underlying causes of malnutrition, enhancing the effects of any individual intervention. Finally, the area of study has suffered an important food crises in the last two years and thus population is at high risk of food insecurity and with increased potential to benefit from the interventions proposed.

We have assumed an intracluster correlation coefficient (ICC) of 0.006 based in the literature [21], and computed the design effect and inflation factor as follows:

Inflation factor = 1 + [(m– 1) x ICC]

where m is the size of the participants to be included in each cluster thus,

Inflation factor = 1 + [(40–1) x 0.006] = 1 + [39x0.006] = 1 + 0.234 = 1.234

The 15% was added as expected follow-up loss rate (estimated based on perinatal mortality, 50.2 per 1000 live births, according to World Bank data^1^).

The study was conceived as a community trial, controlled, open, non-inferiority, randomized by clusters.

Thus, it’s necessary to recruit 12 clusters per each arm with 40 subjects per cluster to achieve 80% power to detect a difference of 0 when the non-inferiority difference is 0.098. The proportion in the experimental groups (SoC alone or SoC + CT) is assumed to be 0.475 under the null hypothesis and 0.377 under the alternative hypothesis. The proportion in the comparator group (SOC + SN) is 0.377.

In all cases the statistic test used is the one-sided Likelihood Score Test (Farrington & Manning). The intracluster correlation is 0.006, and the significance level of the test is 0.025.

**Fig. 2:**
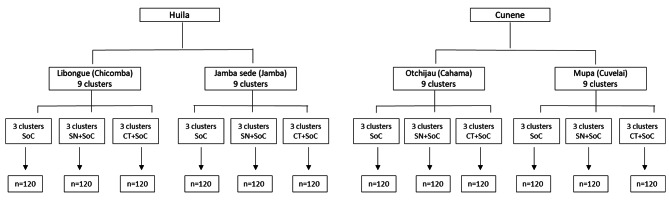
Clusters allocation and expected sample size

In Table 1 and in Fig. 2 we show the distribution of the clusters.

**Table 1:** Participants distribution by trial arm

Intervention	Clusters * Participants	Total
Standard of care (SoC)	12*40	480
SoC + Nutritional supplementation	12*40	480
SoC + Cash transfer	12*40	480
**Total**	**1440**	**1440**

### Study clusters

Study clusters refer to villages or neighbourhoods (both referred in this text as *communities* interchangeably). Being that we need to recruit 40 pregnant women per cluster, we calculated the necessary population size of the cluster with the 3,72% pregnancy prevalence provided by local health authorities^2^ and that calculation resulted in a cluster size of around 1075 people.

We worked with local authorities to identify the villages and neighbourhoods that fulfilled the inclusion criteria. These were listed, along with their estimated populations, and were geographically located in maps elaborated by the project. We then elaborated a list of eligible clusters by: (a) identifying as clusters those communities with a population size around 1075, (b) subdividing larger villages and neighbourhoods into smaller population units of around 1075 people and identifying them as clusters and (c) grouping small and proximate villages to compose additional clusters of the required 1075 population size.

The study design was randomized by clusters with the three intervention arms grouped by *communes* thus; we had separate clusters’ lists for the communes and in each of the *communes* we displayed a public ceremony with local administration and community leaders. The names of all potential clusters within the *commune* were written in folded pieces of paper and placed in a lottery ballot, from which they were taken randomly to guarantee concealment.

Once the clusters to participate in the study were selected, we proceed with the recruitment and random allocation of clusters to the different arms of the study.

### Recruitment and random allocation

The recruitment of the clusters was done by sensitisation sessions with the local authorities to whom the study design and planned interventions were explained in detail.

The local authorities (named *Sobas* and *Seculos* in Angola) had to sign an informed consent to allow for the participation of their communities in the study, before knowing to which arm their community was going to be allocated.

After signing the informed consent, a community leader representing the cluster had to take a slip of folded paper from a lottery ballot box where each folded paper contained the name of one intervention arm. The cluster of the community leader picking up the paper was then allocated to the intervention arm written in the paper. This way allocation concealment was guaranteed. In each commune, three clusters were allocated to each of the intervention arms.

For the recruitment of participants, the week before the first field visit, local ADECOS conduct an active search to identify the pregnant women in the community. They visit the households to inform and sensitise all members of the family as well as pregnant women about the study to take place and the need of their participation in the upcoming survey visit. If ADECOS feel reluctance or uneasiness in the household about being part of the study, they approach the community leader and request his mediation to sensitise the family to the objectives of thestudy.

The allocation of participants was done by complete enumeration. Up to 40 women living in the selected cluster were recruited by order of identification by the ADECOS in the previous weeks, and receive the intervention allocated to their cluster. Women were requested informed consent to participate in the study after informing them about the study overall and about the arm of the study they had been allocated to.

### Eligibility criteria

#### Inclusion criteria

*For municipalities*.

- Be considered as one of the municipalities prioritized by the FRESAN program;
- Have a multidimensional poverty level of 4 or 5 according to the Angola National Statistical Institute classification;
- Acceptance by local and traditional authorities.

*For communes*.

- Do not have community nutritional interventions in place or forecasted at the time of inclusion.

*For communities [Villages (aldeias) and neighbourhoods (bairros)]*

- Do not have other interventions of monetary or nutritional transfers (*nutrition-specific* or *nutrition-sensitive*) in place or forecasted at the time of the inclusion in this study;
- Acceptance by local authorities through the informed consent form signed by the community leader;
- Place accessible by a 4 × 4 vehicle (especially during the rainy season);
- Have a reference health post with reference health personnel.

*For participants*.

- Women 16 years and above;
- Pregnancy confirmed by pregnancy test;
- Acceptance to participate in the study through the free and informed oral consent form confirmed and signed by a research investigator;
- Live newborns of the participant pregnant women;

#### Exclusion criteria

*For participants*.

- Women 16 years of age or above, pregnant, who do not belong to pre-selected neighbourhoods/villages;
- Women who don´t live in the household in a regularly manner;
- Women who have planned to travel or move out of the neighbourhood within the follow-up period of the study;
- Women who express the impossibility of attending the follow-up visits;
- Women with a history of alcohol abuse (considered as intake of > 3 drinks on any day or > 7 drinks per week);
- Any condition that may affect the intervention/follow-up compliance (at the discretion of the investigator);
- Pregnant women with acute malnutrition (arm circumference < 21 cm).

### Outcome measures

The outcomes are measured at individual level, but analysis and interpretation will be made only at cluster level.

#### Primary outcome

- Proportion of children with stunting (HAZ< -2 below the reference median) at 24 months of age.

#### Secondary outcomes

- Child mortality rate at 3, 6, 12, 18 and 24 months of age.
- Proportion of neonatal low birth weight, and low birth weight for gestational age.
- Proportion of children with anaemia (Hb < 12 g/dL) at 6, 12, 18 and 24 months of age.
- Cumulative incidence of morbidity (mainly malaria, diarrhoea and pneumonia) in children below 2 years of age at 3, 6, 12, 18, and 24 months of age.
- Primary household caregiver’s knowledge, attitudes and practices related to perinatal and children’s caring practices including breastfeeding and hygiene and sanitation at baseline and endline.
- Women and children minimum dietary diversity at baseline and endline, and at 3, 6, 12, 18 and 24 months of age of the participant child.

### Measurements

#### Data collection tools

##### Questionnaires

At baseline and end line, we use self-elaborated questionnaires to collect general information of the household, as well as specific information of the head of the household, the primary household caregiver and the participant pregnant women. Sections in these questionnaires include: sociodemographic data, household assets, food security, knowledge, attitudes and practices related to children’s caring practices as well as hygiene, and sanitation, women’s empowerment, dietary diversity at household, women and children’s level, perinatal care and pregnant women’s morbidity and mental health.

For the food security section we used the FAO standardized survey modules on household dietary diversity score [22] and on the food insecurity experience access scale (FIES) survey module [23]. To measure women’s dietary diversity we used the Minimum Dietary Diversity for Women questionnaire [24] adapted to Angola by the Global Diet Quality Project^3^, and to assess children’s dietary practices we used the standardized survey modules provided by the World Health Organization and UNICEF [25].

In the follow up visits, data will be collected from study participants with specific self-elaborated questionnaires (mothers and children’s questionnaires).

Original questionnaires are in Portuguese and translation to local languages was conducted during the training with enumerators.

The questionnaires are in digital format, designed with the Ennov Clinical Software, and data is collected with the application Ennov Clinical through smart devices run on apple-based platforms. Data is sent to the data management centre every few days upon internet coverage availability.

#### Anthropometry

Arm circumference is measured in pregnant women and children using standard MUAC bracelets.

During the survey, women identified with a MUAC below 210 mm are referred to the nearest health facility for standard treatment. Children identified with a MUAC < 125 mm are referred to the nearest out-patient therapeutic centre.

Body weight (kg) and height (cm) are measured in children. Weights are measured using the ADE M321600 electronic floor scale with mother and child weighing function.

Height is measured in children (standing or recumbent) using a wooden board suitable for measuring the length of infants and the height of children up to 160 cm.

#### Biochemical measures

Women identified as pregnant in the community are tested with the DIAGNOS© hCG one-step pregnancy tests to confirm their pregnancy status. If a negative result is obtained the test is repeated, and if a negative result is again obtained, the woman is referred to the health centre for follow up and she is excluded from the study.

Haemoglobin is measured using the *Very-Q RED* haemoglobin monitoring system (0.1 g/dl accuracy) in the participant women during visits where she is pregnant and in participant newborn children at age 6 months, 12 months, 18 months and 24 months. If any participant is identified with an Hb < 5 g/dl is referred to the nearest public health facility.

Malaria infection is measured with *Abbot Malaria Ag P.f/Pan* rapid test in children at 6, 12, 18 and 24 months of age. Children identified with a positive result are referred to the nearest health facility for standard treatment.

#### Physical examination

Nursing and medical students perform a physical examination of participants and follow a standardized procedure of health care indications and referral to health facilities for any danger sign observed, including an Hb < 7 g /dL with symptoms.

### Data collection team

There are 12 teams of enumerators supervised by three research investigators and three field supervisors. Each team is composed by one medical or nursing student and one ADECOS and both received previous training for conducting the interviews and entering the data.

A total of 54 ADECOS (out of the 214 recruited by Crescer) participate in the data collection and are trained in survey procedures. The training for data collection was done to pairs of students and ADECOS as they have different roles to play during the survey implementation. The students are in charge of asking and recording the responses in the tablet, as well as taking the measurements and doing the physical examination. The ADECOS are responsible of introducing the team to the families and acting as translators during the interviews, as well as supporting with the measurements when required.

Enumerators and supervisors were also trained in anthropometric and biochemical measurements according to international recommendations, as well as in the standard operational procedures elaborated for the data collection activities.

Pilot studies were conducted as part of the field work training and to test the tools in both Huila and Cunene provinces.

### Data collection timeline

The recruitment and baseline survey has started in 10 October 2022 and will continue until March 2023, thus recruitment is ongoing at the time of submission of this protocol for publication. The research team makes field visits every 3 months in order to collect baseline measurements on all study households (household questionnaire) and participants (pregnant women), and follow up data on participants (mother and child questionnaires) until the newborn child turns 2 years of age (end line survey). Thus, the end line surveys will be conducted at different times, depending on the date and the gestational age of the women at the time of recruitment, with dates for end line surveys ranging between November 2024 andFebruary 2025.

The SQ-LNS Supplementation and the unconditional cash transfers are implemented during the quarterly visits. See Table 2.

**Table 2:** Data collection and interventions timeline

Data collection and interventions	Recruitment and baseline	Follow-up visits every 3 months	Endline
**Dates**	From October 2022 to February 2023	From October 2022 to July 2025	From November 2024 to August 2025
**Data collection**			
Household questionnaire	x		x
Mother questionnaire	x	x	x
Child questionnaire		x	x
**Interventions**			
Nutrition supplementation	x	x	
Unconditional cash transfer	x	x	

Depending on the gestational age of the pregnant women and the age of the child at the time of the visit different data will be collected. Women will complete one or two questionnaires during pregnancy (around 6th month of pregnancy and/or around 9th month of pregnancy). In addition, once the children are born, all women and newborn children included in the study will be surveyed (mother and child questionnaires) at 3 months, 6 months, 12 months, 18 months and 24 months of age of the children with a precision range of 45 days. Outcomes to be collected at those time points are detailed in Table 3.

**Table 3:** Data collected and time points according to the women’s gestational age and newborn children’s age

Data collection	Questionnaire	Recruitment and baseline	Follow-up visits						Endline
			Pregnant women gestational age		Children’s age				
			6 months	9 months	3 months	6 months	12 months	18 months	24 months
Participants informed consent		X							
Sociodemographic data	HQ	X							X
Household characteristics and assets	HQ	X					X		X
Household food security	HQ	X					X		X
Household WASH practices	HQ	X					X		X
Knowledge, attitudes and practices (KAP) related to children’s caring practices and WASH	HQ	X							X
Household mortality	MQ	X	X	X	X	X	X	X	X
Pregnancy and prenatal care data	MQ	X	X	X					
Medical history	MQ	X							
Delivery and postnatal care data	MQ				X				
Concomitant medication	MQ	X	X	X	X	X	X	X	X
	CQ				X	X	X	X	X
Physical examination	MQ	X	X	X					
	CQ				X	X	X	X	X
Anthropometry	MQ	X							
	CQ				X	X	X	X	X
Laboratory examinations	MQ	X	X	X					
	CQ					X	X	X	X
Relevant clinical events	MQ		X	X	X	X			
	CQ				X	X	X	X	X
Relevant social events	MQ	X	X	X	X	X	X	X	X
Morbidity	MQ	X	X	X					
	CQ				X	X	X	X	X
Mortality	MQ		X	X	X	X	X	X	X
Dietary practices	MQ	X	X	X	X	X	X	X	X
	CQ				X	X	X	X	X
Malaria profilaxis	MQ	X	X	X					
Deworming	MQ		X	X					
	CQ						X	X	X
Vitamin A supplementation	CQ					X	X	X	X

WASH: Water, hygiene and sanitation; HQ: Household questionnaire; MQ: Mother questionnaire; CQ: Children questionnaire

### Planned statistical methods

The analysis will be done only at cluster level. Descriptive analyses will be carried out in all independent variables at cluster level to characterize groups at baseline and identify potential differences between them.

Categorical variables will be presented as frequencies and percentages and continuous variables as medians (interquartile range) or means (standard deviation) according to data distribution.

Distribution normality will be checked by means of histograms and the Kolmogorov-Smirnov test.

The chi-square test or Fisher’s exact test will be performed to compare frequencies between categorical variables and the Mann-Whitney test or Student’s t-test will be used to compare continuous variables.

For the analysis of effectiveness, the comparison of the proportion of participants with stunting between the clusters will be performed through generalized linear models with log-link and binomial distribution. We will estimate the value of the difference between the experimental interventions (SoC, SoC + CT arms) and the comparator intervention (SoC + NS, arm).

We will do the analyses in two study populations. The one defined per intention to treat (ITT), comprises all randomized participants including those that did not fulfil the inclusion criteria but were randomized, those with an early withdrawal before starting the interventions and those facing major protocol deviations, all of which are considered treatment failures. The other one, obtained per protocol (PP), is the subset of participants belonging to the ITT who do not deviate from the protocol. Thus, it excludes participants facing any deviation from the protocol (do not comply with the instructions of the interventions or interrupt or permanently modify the proposed scheme).

Significance will be set at *p* < 0.05 level.

## Discussion

The complex and multi-causal nature of child stunting makes especially challenging the endeavour of its prevention and reduction. The range of potential interventions and the financial constraints to implement them all, results in decision takers and policy makers facing the difficult task of choosing one strategy over others. The justification of these selections should be based on impact and sustainability of the interventions at hand.

Thus, generating high-level evidence on the effectiveness of different type of interventions on the prevention of child stunting, and understanding the pathways by which they operate can contribute to the uptake of better-informed decisions, and better design of future strategies.

There are potential difficulties of implementing randomized control trials to test the effectiveness of public health interventions which have complex causal chains and are subject to effect modification in different populations. Many of them relate to the feasibility and ethics during implementation and the complexity of assuring internal and external validity of results obtained [26].

The randomization of clusters rather than individuals facilitates trial recruitment, reduces the costs of research, and may improve internal validity by reducing the risk of contamination [27]. However, in this case, it also implies that participants know the arm to which they are allocated before consenting to participate in the study and this can introduce a bias towards a lower participation in the less preferred intervention arm, the standard of care alone. Although this bias is difficult to prevent, the project will collect rates of refusal and drop out in order to assess the likelihood and magnitude of its occurrence, to consider it in the analysis and interpretation ofresults.

The Crescer consortium dedicated eighteen months to the preparation of the MuCCUA study. Only remote and difficult to access communities fulfilled inclusion criteria of having no intervention in place so many field trips were necessary to locate the communities, and it was necessary to elaborate timely maps for future visits, as there were no roads or maps to access them. Selected communities were visited several times to conduct sensitisation sessions about the project, obtain informed consent from community leaders and recruit the community agents (ADECOS) to implement the standard of care programme. Having the Angolan Fundo de Apoio Social as partner in the consortium made the recruitment of communities feasible, as they have a long history of community work in the study area.

The Crescer consortium participates both in the data collection of the study and in the implementation of the interventions. However, only few actors actively participate in the two activities (54 ADECOS out of the 214 ADECOS recruited for the project and the researchers that supervise both data collection and interventions). As enumerators and supervisor codes are entered in the questionnaire we will conduct sensitivity analysis to test if the fact of having same teams implementing interventions and collecting data has any impact in the data, although all teams participate in the three arms of the trial so this bias may not impact differences found between them.

In relation to the ethical concerns that may arise when implementing RCTs for public health interventions evaluation, the Crescer project assures that an improved standard of care programme is in place in all communities participating in the MuCCUA study, thus providing an overall benefit to all.

In order to preserve as much as possible the internal validity of results, the project collects comprehensive information on baseline characteristics of the interventions and control arms groups during the recruitment and baseline survey. Although external validity is difficult to ensure as context specificities play an important role in the degree of effectiveness obtained, understanding the pathways by which the different interventions effect stunting may provide a starting point framework for evaluating similar interventions in different contexts.

Moreover, one important strength is that the study is targeted to pregnant women and their newborns closely followed by the ADECOS, and consequently, we expect to obtain valuable information on the impact of the different strategies on stunting prevention when targeting the 1000 days, starting during the pregnancy period.

Furthermore, at the time the study was designed the situation of food and nutrition insecurity in South Angola was not as severe as it is now. Nevertheless, this new scenario will give us the opportunity to assess how severe food insecurity and acute malnutrition interact with stunting progression and the impact of a long run project with a chronic malnutrition objective within humanitarian crises as the one lived today in these regions of Angola.

## Data Availability

The datasets used and/or analysed during the current study are available from the project coordinator, Israel Molina Romero, on reasonable request at israel.molina@vallhebron.cat.

## Footnotes

1 Mortality rate, infant (per 1,000 live births) - Angola | Data [Internet]. [citado 9 de julio de 2021]. Disponible en: https://data.worldbank.org/indicator/SP.DYN.IMRT.IN?locations=.

2 Municipio da Jamba. Distribuição da população por area sanitaria/2021.

3 Available at: https://www.dietquality.org/.

## Declarations

### Data monitoring and auditing

Monitors will conduct regular monitoring visits, during which they will inspect and verify the source data of Informed Consent Forms, medical records, laboratory results, and protocol deviations. Monitoring visits will be made periodically by VHIR representatives or designated monitors to ensure compliance with Good Clinical Practice (GCP) and all aspects of the protocol. Source documents will be reviewed for consistency with data in the electronic Case Report Forms (CRFs) and any data clarification forms/consultations.
